# Experience of using an open source clinical trials data management software system in Kenya

**DOI:** 10.1186/1756-0500-7-845

**Published:** 2014-11-26

**Authors:** Moses M Ngari, Naomi Waithira, Roma Chilengi, Patricia Njuguna, Trudie Lang, Greg Fegan

**Affiliations:** KEMRI-Wellcome Trust Research Programme, Centre for Geographic Medical, Research Coast, PO Box 230, Kilifi, 80108 Kenya; Center for Infectious Disease Research, Lusaka, Zambia; Centre for Tropical Medicine, Nuffield Department of Medicine, University of Oxford, Oxford, UK

**Keywords:** Clinical trials, Data management, Open-source systems, OpenClinica

## Abstract

**Background:**

Clinical trials data management (CTDM) remains one of the many challenges in running state of the art trials in resource-poor settings since most trials do not allocate, or have available, sufficient resources for CTDM and because of poor internet connectivity. Open-source software like OpenClinica could be a solution in such scenarios.

**Findings:**

In 2007, the KEMRI-Wellcome Trust Research Programme (KWTRP) adopted OpenClinica (OC) community edition, an open-source software system and we share our experience and lessons learnt since its adoption. We have used OC in three different modes; direct remote data entry from sites through Global System for Mobile Communications (GSM) modems, a centralized data centre approach where all data from paper records were entered at a central location and an off-line approach where data entry was done from a copy of database hosted on a field-site server laptop, then data uploaded to a centralized server later. We have used OC in eleven trials/studies with a cumulative number of participants in excess of 6000. These include large and complex trials, with multiple sites recruiting in different regions of East Africa. In the process, we have developed substantial local capacity through hands-on training and mentorship, which we have now begun to share with other institutions in the region.

**Conclusions:**

Our experience demonstrates that an open source data management system to manage trials’ data can be utilized to international industry standards in resource-poor countries.

## Findings

### Introduction-the need for open-source software

Clinical trials are primarily set up to answer specific research questions; answering these research questions entails gathering, storing and processing data. There is a need to develop stringent strategies of collecting, managing and producing high quality trial data [1]. Data management is a complex process that involves investigators collecting data directly from the trial participants, laboratory technician running trial samples, data entry by data entry clerks, data monitoring, system administrators maintaining the data within the database and communicating with trial managers, and analysis by statisticians and study Investigators. Given this complexity, few trials go exactly as initially planned; in the course of the trial, case report forms (CRF) may need updating, a new trial site may be added and new technology may emerge. With many clinical trials going global [2], managing all these issues and stakeholders to yield data of high quality needs a well thought-out plan. All these complexities notwithstanding, an investigator has to worry about the cost of acquiring, installing and maintaining a data management system and compliance with set guidelines and standards [3].

Historically, trials’ data have been captured on paper and investigators had to handle large volumes of paper. However, electronic data capture (EDC) and use of electronic case report forms (eCRF) are becoming more utilized and have revolutionized the way trial data are managed [4–6]. The advent of EDC and eCRF has reduced the burden of organizing paper CRFs, greatly reducing the time to avail data in electronic formats and improved the efficiency in running clinical trials [7]. However, these benefits come with some challenges. The whole process of clinical trial data management using EDC and eCRF requires substantial capital investment and utilizing sophisticated technology requiring highly trained professionals [8, 9]. Industry regulations require EDCs that are validated to ensure trial data reliability, accuracy, security and electronic audit trails that document every action on the EDC [10]. In the rapidly changing information technology environment, careful consideration is needed before investing in new hardware or software. And, any EDC system comes with the additional challenges of installation, customization, configuration or/and integrating with existing systems such as laboratory or clinical surveillance systems. For academic institutions, small-size research institutions, individual investigators and poorly funded research institutions, particularly from developing countries, traditional paper based approaches with data entry at a central location may be the most feasible considering all these factors.

The demand to make clinical trials management affordable whilst still complying with industry standards, regulatory requirements and best practices continues to grow. There is need to build conducive environments that supports flexible trial design, configuration and quick deployment of robust EDC, real-time data capture, extraction, analysis and reporting of trials’ findings [10]. This has led to adoption of EDC and e-clinical systems in clinical trial data management in various research environments with varying degree of success [11]. We have previously argued [9] that an open source clinical trial data-management system could help in achieving the above targets. Within a short time, the usage of open source EDC has grown rapidly and, a good example is OpenClinica (OC) [12]. Adoption of OpenClinica as a clinical trials’ EDC has grown tremendously since its release in 2005, currently boasting of over 15,000 community members with a presence in over 100 countries [12]. Open source EDCs are becoming common because they are free to download and use, come with low/free cost of maintenance and are easy to install and use. They can be customized to the requirements of the end-users since their codes are available freely, can be configured to the security standards of the user and are interoperable with existing systems. Open source EDCs have the potential of increasing and improving public health research activities and raising academic standards because of their availability (they are inexpensive/free) and have a community of users and developers, where experience and ideas are freely exchanged [13–15]. Such exchanges enrich and reify the source code, improving the EDC’s quality and leading to additional useful features to the users.

In this article we describe our experience with OpenClinica, an open-source EDC in managing clinical trials data in Kenya and the surrounding region.

### How we started

A Clinical Trials Facility (CTF) was setup within KWTRP because there was a need to have a coordinating centre for multiple trials and develop capacity to manage trials to standards used in the developed world. For the last 25 years, KWTRP has conducted clinical, laboratory and epidemiologic research within Kilifi County and its environs [16]. There was a wide difference between academic and pharmaceutical or product development trials, and so the aim was to ensure that all studies had access to monitoring, good data management and trial coordination. The CTF was set up to provide a centralized resource of study support functions in order that skills and best practice could be shared between different studies and disease areas. This worked very well, especially for data management where a dedicated data management office was set up. After careful consideration of resources needed, time, human capital and international good clinical practice (GCP) standards, we chose OC as our Clinical Trial Data management System (CTDMS) [9]. This decision was followed by a one week on-site training by the developers of OC, Akaza Research LLC, in mid 2007. No external system validation was done, however, we internally validated the initial version to meet the GCP requirements, and have continuously validated all updated versions before use. Later an in-house ‘OC group’ was set up to champion and oversee implementation of the system. The group comprised the heads of CTF and Statistics department, data managers, a system administrator and other stakeholder (lead PI). The group held regular meetings through which targets were set, learning tasks appointed, experiences shared and progress reviewed.

Up to the present date, we have maintained a system administrator offering technical support to the users while individual trial instances are designed and managed by specific data managers. The initial system administrator had a Bachelors level degree in Computer Science with skills in programming and databases and worked within the ICT department spending no more than 5% of their time on OC. The data managers come from a diverse range of academic backgrounds (Statistics, IT, Biological Sciences) and are hired directly for specific trial teams but sit as a pool within CTF allowing for sharing of ideas and experience, thus fostering consistency in how KWTRP runs trials.

### Our computing environment

We configured OC on a server running Windows Server 2003 operating system and using an Apache Tomcat application server and a PostgreSQL database. In line with KWTRP IT standards, the software and database have been installed on separate servers to ensure greater security, manageability, and flexibility. To add further data security the OpenClinica software and PostgreSQL database were integrated with the Programme’s Microsoft Active Directory based, central user access and management system.

These systems were installed within the Programme’s ‘private cloud’ infrastructure that is based on Microsoft Hyper-V virtualisation, HP Blade hardware, NetApp storage, and within our Internet Service Provider (ISP)’s wide area network. Implementing the solution within this environment provides the following features; database backup every 15 minutes, ability to deliver the service from either Kilifi or Nairobi office, ability to recover the service anywhere in the cloud within 30 minutes. However, one is able to run OC on a much smaller specified environment, which meets the following criteria as specified by the developers, Akaza Research LLC in Table 1[12].

**Table 1:** **OpenClinica system requirements adopted from the developers (** https://docs.openclinica.com/installation/system-requirements **)**[12]

Version	Server OS	Database	Application server	Language	Memory	Disk	Client browser
3.1	Windows 2003	Postgres 8.4 (recommended)	Apache Jakarta	Java Development Kit (JDK) 6	256 MB Minimum	500 MB Minimum	Internet Explorer (IE) 7 or 8
	Red Hat Enterprise		Tomcat 6.0.32		2GB (Recommended)	2GB (Recommended)	Firefox 3.0+
	Linux 4.0						
	CentOS Ubuntu	Oracle 10					
3.2	Windows 2003 R2	Postgres 8.4	Apache Jakarta	JDK 6 or JDK 7 (Recommended)	256 MB minimum	500 MB Minimum	IE 11 Firefox 25
	CentOS 6.5 (~Red Hat Enterprise Linux 6.4)		Tomcat 6.0.32 or 7.0.42 (Recommended)		2GB Recommended	2 GB Recommended	
	Ubuntu 12.04 LTS						
3.3	RHEL/CentOS 6.5 or above 64-bit (recommended)	Postgres 8.4	Apache Tomcat 7.0X	JDK 7	2 GB	2 GB	IE 11+ Firefox 25.01
	Windows 2003 R2 or above 32-bit						
	Ubuntu 12.04 LTS 64-bit						
3.4	RHEL/EntOS 6.5 or above 64-bit (recommended)	Postgres 8.4	Apache Tomcat 7.0X	JDK 7	2 GB	2 GB	IE 11+ Firefox 25.01
	Windows 2003 R2 or above 32-bit						
	Ubuntu 12.04 LTS 64-bit						

### System security

Security in OC is ensured through use of usernames and passwords to authenticate users and provision of different privilege levels to users based on their roles that are specified by a system administrator. For example clinical monitors and data specialists (statisticians/data analyst) have read only rights; they cannot edit data in the database. Additionally the system requires the investigator or designated member to electronically sign the participants’ records data before database lock. Such electronically signatures are legally binding and make the investigators take full responsibility of ensuring the electronic data are complete and accurate.

Within KWTRP, we implemented additional security by installing the database and the application on different servers. A demilitarized zone (DMZ) was created for further security since the OpenClinica server was required to be publicly accessible. The DMZ is a logical sub-network in which publicly accessible servers are placed. This ensures that in case the publicly accessible server is compromised, the attacker is not able to reach other servers in the internal network. We also implemented SSL (Secure Socket Layer), a security protocol that ensures data submitted on the internet is encrypted and secure from eavesdropping.

### Our successful implementation of different OC modes

For the last five years that we have been using OpenClinica, we have run eleven clinical trials ranging from phase I-III, involving in excess of 6000 study participants. We have used it in three different modes; direct remote data entry from sites using GSM modems, a centralized data centre approach where facsimiles of all paper records were sent and data entered and an off-line approach where data entry was done from a copy of database hosted on a field-site server installed on a laptop and the data uploaded on the live, data centre located server later, at the end of each day.

Technically, we have been able to install and run OC within our computing environment without having to incur extra expense of new software. Being a web-based system, we have used it extensively to run our multisite studies with the database hosted at our centre in Kilifi and being accessed for data entry, monitoring and extraction from our various sites or collaborators’ centres. Internet access in Kenya is readily available via GSM modems supported by telecommunication companies either through post-paid or pre-paid tariffs. We have managed a near real-time data entry from the sites of one large multisite study (case study 2) making data available to researchers promptly. The study sites used GSM modems to access the system, meaning we used the internet only during data entry keeping the cost of running the study minimal. Data from one of the study (case study 1) was extracted by a study statistician at the UK’s Medical Research Councils’ Clinical Trials Unit (MRC-CTU) in London. At CTF, we have a pool of clinical monitors electronically monitoring various studies using OC, these include studies from East Africa region that we offer clinical monitoring services. Most of these studies’ data are accessed remotely at CTF by the clinical monitors. This way, we have been able to manage near real-time data entry from remote sites, made data available to a statistician remotely from distant parts of the world and offer electronic clinical monitoring to many studies within the region.

Where we conducted trials in areas with erratic power supply or internet connectivity, we adopted an offline strategy as had been done by others in such settings [17]. A replica of the main OC database running on the server at Kilifi centre was installed on one laptop which acted as a “field server”. The field server was configured to create a wireless network to connect with two data entry client laptops in the field. Data entry was then done from the client laptops with the data being saved on the field server. Automatic backups to an external hard disk, was set to run after every two hours. At the pre-set timepoints, a database dump script was ran on the field server to produce a database file which was then copied to the live server at KWTRP data centre.

#### Studies

Table 2 details the studies that we have carried out using the OC platform. The way these have been implemented using this system varied given limitations or constraints of available technology, staff and connectivity in the sites where we implemented these studies. We highlight three use cases based on studies that used different modes of using OC as shown in Table 3.

**Table 2:** **Inventory of studies carried out to date using OpenClinica**

Study name	Dates	Registration in trials registry hyperlink	Number of sites & study participants	Related publications
Methotrexate	Mar–Aug 2009	NCT00791531	1 site	Chilengi et al. [20]
		http://clinicaltrials.gov/show/NCT00791531	25 participants	
FEAST	Jan 2009-Jan 2011	ISRCTN69856593	6 sites	Maitland et al. [21]
		http://www.controlled-trials.com/isrctn/pf/69856593	3141 participants	
MODMAL*	Apr – Oct 2009	NCT00890695	1 site	
		http://clinicaltrials.gov/ct2/show/NCT00890695	400*	
CTX	Nov 2009 – Mar 2014	NCT00934492	4 sites	
		http://clinicaltrials.gov/show/NCT00934492	1781 participants	
PUFA/Njugu Plus	May2012-May2013	NCT01593969	1 site	
		http://clinicaltrials.gov/ct2/show/NCT01593969	60 participants	
TRAP VAC	Jun 2010-May2011	NCT01379430	1 site	Ogwang C et al. [22]
		http://clinicaltrials.gov/show/NCT01379430	30 participants	
VAC 046	Mar-Aug 2012	NCT01666925	1 site	
		http://clinicaltrials.gov/ct2/show/NCT01666925	120 participants	
CATMAP	Apr 2011-Nov 2012	NCT01190371	1 site	
		http://clinicaltrials.gov/show/NCT01190371	175 participants	
MESALAMINE	June2013-April 2014	NCT01841099	1 site	Jones et al. [23]
		http://clinicaltrials.gov/show/NCT01841099	44 participants	
EAPHLNP	April-July 2013	NCT01899820	7 sites	
		http://clinicaltrials.gov/ct2/show/study/NCT01899820	352 Participants (Kwale Site only)	
MALPAC	Oct2013-Feb2014	PACTR201309000625311	1 site	
		http://www.pactr.org/ATMWeb/appmanager/atm/atmregistry?_nfpb=true&_windowLabel=basicSearch_1_2&basicSearch_1_2_actionOverride=%2Fpageflows%2Ftrial%2FbasicSearch%2FviewTrail&basicSearch_1_2id=625	90 participants	

*Stopped early due to lack of study participants only 64 recruited of 400 target.

**Table 3:** **Details of exemplar case studies**

Study name & approximate database size (Mb)	Description data of distinct CRF (# of data items)	Number of events	Method of OC deployment
FEAST (32)		10 events	Data centre approach that collated, entered and managed all inputs from six study sites at centralised location
	Enrolment (29)	Enrolment (×1)	
	Clinical information (243)	Clinical information (×2)	
	Additional Assessments (74)	Additional assessments (×3)	
	Serious Adverse Events (31)	Serious Adverse Events (×1)	
	Follow up (34)	Follow up (×3)	
CTX (12)		7 events	Data entered into the centralised system directly from the four study sites
	Enrolment (71)	Enrolment	
	Discharge Information (21)	Discharge	
	Follow ups (198)	Follow ups (×9)	
	Study conclusion (12)	Study conclusion	
	Adverse Events (13)	Adverse Event	
	Serious Adverse Events (14)	Serious Adverse Event	
	Concomitant medication (28)	Concomitant medication	
TRAPVAC 046 (5.5)		26 events	Field based laptop server with two data entry clients
	Screening (119)	Screening	
	Pre-vaccinations check (80)	Pre-vaccinations (×2)	
	Vaccines administration (20)	Vaccine administration (×2)	
	Systemic Adverse Event (126)	Systemic Adverse Event (×2)	
	Follow ups (180)	Follow ups (×6)	
	Concomitant Medication (7)	Concomitant medication	
	Adverse Event (15)	Adverse Event	
	Serious Adverse Event (7)	Serious Adverse Event	
	End of study (3)	Unscheduled Visit	
		End of study	

#### Case study 1: FEAST (Fliud Expansion as a Supportive Therapy)

FEAST was a randomised, open-label clinical trial that tested the safety and efficacy of giving rapid fluid resuscitation compared with no bolus (control) in severely ill hospitalized children (ISRCTN: 69856593). The trial was conducted in six hospitals in East Africa: four hospitals in Uganda and one hospital in each of Kenya and Tanzania, running from January 2009 to April 2011. Paper CRF elements were transcribed from source documents and 100% study monitoring/verification done for a subset of selected data items. Due to limited internet connectivity at some of the sites, data entry could not be done directly, thus paper CRFs were sent by courier service to the central data centre in Kilifi from where data entry was done. Data queries were resolved through data query forms sent by the data manager to the study sites through email. The data was later extracted for analysis by a statistician based at the UK’s Medical Research Councils’ Clinical Trials Unit (MRC-CTU) in London. The study result was published in 2011 [21].

#### Case study 2: CTX (Cotrimoxazole prophylaxis)

CTX was a randomised, double blind, placebo-controlled trial with the primary objective of determining the efficacy of cotrimoxazole prophylaxis in reducing post-discharge mortality among hospitalized, HIV-uninfected but severely malnourished children (NCT00934492). The trial recruited participants from four district hospitals distributed across Kenya with three sites at the Coast and one in Nairobi. OC was setup and managed at the Kilifi data centre. At least two field workers (field staff with secondary education) were trained to enter the data at each site and the application was accessed directly from sites using GSM modems connected to the internet. Each site only had access to their respective data and data entry was done at near real time once the paper CRFs had been completed. Study data was automatically extracted once a week, and imported into Stata (Stata Corp, College Station, TX USA). Data quality was ensured through resolution of queries raised by electronic monitoring, having 100% source data verification, validation checks set in the database and checks run on the extracted data. The study completed data collection at the end of March 2014 but is yet to publish results (expected late 2014).

#### Case study 3: TRAPVAC 046 (Thrombospondin-related adhesion protein vaccine 46th trial)

TRAPVAC 046 was the locally used acronym for a single blinded controlled phase IIb trial that assessed the efficacy of a heterologous prime-boost vaccine strategy with ChAd63 ME-TRAP and MVA ME-TRAP in healthy adults in Kenya (NCT01666925). This small study, with a sample size of 120 participants, recruited healthy men, 18–50 years old, residing within Kilifi county where KWTRP has its’ headquarters. Data entry was done from paper CRFs directly at the field site by both clinicians and data entry staff on laptops connected to the field server. A wireless local area network connected the additional laptops to the field server. At the end of each day, a copy of the database on the field server was uploaded to the main OC server in the data centre. Electronic monitoring and data extraction were done from this main/centralized OC server by the study monitors and statistician respectively. The study completed data collection but is yet to publish its results.

### Capacity strengthening

We have held training within and outside Kenya and hosted a number of data managers from other institutions interested in learning and using OC as shown in Table 4. Most recently we have been consulted by investigators of an Ethiopian intervention study against Podoconiosis [18] starting in 2014 for trial management, statistics, data monitoring and management using OC. We have participated in several workshops and conferences presenting our experience using OC, including the OpenClinica Global Conference of 2010 and sixth European & Developing Countries Clinical Trials Partnership (EDCTP) Forum in Addis Ababa, Ethiopia. The data management group in KWTRP actively participates in the Global Health Trials (http://www.globalhealthtrials.org) which is a free resource to support clinical trials conducted in resource limited settings. Through this we have supported and advised many diverse groups in the use of OC and other data management queries. Amongst other things, we provided training to fellow researchers from the East African region working at Amnauer Hansen Research Institute, Ethiopia, Uganda Viral Research Institute, Uganda and Mwanza Intervention Trials Unit, Tanzania (Table 4).

**Table 4:** **Details of trainings conducted**

Institution/group	Dates	Details
Annauer Hansen Research Institute, Ethiopia (2 pers) and MRC/UVRI Uganda (1 pers), 3 data managers	May to July 2012	The trainees spent two months at KWTRP learning from experienced system users.
Mwanza Intervention Trials Unit (MITU), 1 data manager	March to June 2011	The trainee spent four months at KWTRP learning from experienced system users.
MRC/UVRI, statistician and data management staff with staff from MITU also attending (approx 10 participants)	February 2011	A one week training covering full details of installation and system use.
KWTRP, data entry staff	November 2010	A one day refresher training for data entry staff.
KWTRP, internal monitors	March 2010	A one day training of trial monitors to conduct electronic monitoring.

### Challenges and future plans

Due to the web-based nature of OC, remote access to the database requires a network connection. This poses a challenge for real time data entry as some study sites are located in areas with limited or non-existent internet connectivity; however we have found modalities to work around this as discussed in one of the case studies above.

Initial problems of sluggish response times and system timeouts were ameliorated through adjustment of memory allocation to Tomcat (See http://tomcat.apache.org/) and reducing the application logging levels. XML rules were found to adversely affect the application’s response time and therefore other options for data quality assurance such as post data-entry validation using statistical packages and CRF based validation were explored and used. Help and insights to solve such problems was found by referral to OpenClinica online wikibooks-based user manual [19] and other online information shared by the user community, such as the electronic discussions forums.

As technology evolves, we will link OC with other existing system such as the laboratory and clinical data systems at KWTRP. We will extend the “Extract Data” module to be available directly for other statistical packages such as Stata which is the statistical *lingua franca* at KWTRP. We are also keen on using phones and tablet devices for direct data capture. We are yet to discard paper based primary CRFs and do direct data capture at trial sites but aim to do this in future trials.

## Conclusion

Our five years’ experience shows it’s possible to use OC successfully in different trials employing different EDC infrastructures in resource-poor environments. Working as a team involving investigators, trial managers, clinical monitors, data managers, system administrators and statisticians from the onset of the system selection, installation, training and use was a key driver to successful implementation of the OC system in our institution. Adoption of an open-source CTDM system remains a promising solution to data management of multisite trials, especially in low resource settings.

## Availability and requirements

**Project name:** OpenClinica.

**Project home page:**https://www.openclinica.com/

**Operating system(s):** Linux, Windows.

**Programming language:** Java, PostgreSQL/Oracle.

**Other requirements:** Apache Tomcat, Web browser for example Internet Explorer or Mozilla Firefox.

**Any restrictions to use by non-academics:** None (GNU Lesser General Public License).

## Authors’ information

MN is a data manager working with OC system since 2009 at KWTRP CTF and was responsible for the data management of the CTX study. NW has worked as a data manager and systems programmer at KWTRP since 2008 and was responsible for the data management of FEAST. RC and PN have both been former Heads of the CTF. TL initiated the CTF at Kilifi and was its first Head; she is a thought leader in the issues surrounding the running of clinical trials in resource poor areas. GF has been the Programme Statistician at KWTRP since 2003, he first investigated the possibility of using OC in 2007.

## Abbreviations

KEMRI: Kenya Medical Research Institute
KWTRP: KEMRI-Wellcome Trust Research Programme
CTDMS: Clinical trials data management system
OC: OpenClinica
CRF: Case report form
eCRF: Electronic case report form
ICT: Information and communications technology
EDC: Eletronic Data Capture
CTF: Clinical Trials Facility
GCP: Good clinical practice
UK: United Kingdom
MRC-CTU: Medical Research Council clinical trials unit
EDCTP: Europen & Developing Countries Clinical Trials Partnership
XML: Extensible Markup Language.
